# A hierarchical negative-binomial model for analysis of correlated sequencing data: practical implementations

**DOI:** 10.1093/bioadv/vbaf126

**Published:** 2025-06-10

**Authors:** Katarzyna Górczak, Tomasz Burzykowski, Jürgen Claesen

**Affiliations:** Data Science Institute, Hasselt University, Hasselt 3500, Belgium; Open Analytics, Antwerpen 2600, Belgium; Data Science Institute, Hasselt University, Hasselt 3500, Belgium; Department of Biostatistics and Medical Informatics, Medical University of Bialystok, Bialystok 15-089, Poland; International Drug Development Institute (IDDI), Ottignies-Louvain-la-Neuve 1340, Belgium; Data Science Institute, Hasselt University, Hasselt 3500, Belgium; Department of Epidemiology and Data Science, Amsterdam University Medical Centre, VU Amsterdam, Amsterdam 1081 HV, The Netherlands

## Abstract

Summary

High-throughput techniques for biological and (bio)medical sciences often result in read counts used in downstream analysis. Nowadays, complex experimental designs in combination with these high-throughput methods are regularly applied and lead to correlated count-data measured from matched samples or taken from the same subject under multiple treatment conditions. Additionally, as is common with biological data, the variance is often larger than the mean, leading to over dispersed count data. Hierarchical models have been proposed to analyze over dispersed, correlated data from paired, longitudinal, or clustered experiments. We consider a hierarchical negative-binomial model with normally distributed random effects to account for the within- and between-sample correlation. We focus on different software implementations to allow the use of the model in practice.

## 1 Introduction

High-throughput sequencing methods, such as RNA-seq, are nowadays used in experiments with complex designs that lead to over dispersed and correlated count-data. These data often arise from matched samples, or repeated, or longitudinal measures of the same subject. Analysis of such data should take the overdispersion and correlation into account.

Standard methods developed for RNA-seq data analysis typically rely on either generalized linear models (edgeR [Robinson *et al.* 2010], DESeq2 [Love *et al.* 2014]), where the response variable is modeled with negative-binomial distribution, or linear (mixed-effects) models (limma [Ritchie *et al.* 2015]) applied to transformed data. While these approaches are suitable for simple experiments that involve comparisons of groups of independent samples, they are limited when dealing with more complex experimental designs in which data may exhibit additional variability and within- and between-sample correlations (Tsonaka and Spitali 2021). For instance, edgeR and DESeq2 can take into account overdispersion, but they cannot accommodate random effects that could help to capture the additional correlation structure. On the other hand, limma allows inclusion of a single random effect, but estimates a single genome-wide variance term neglecting the fact that the contribution of the random effect may vary from gene to gene (Hoffman and Roussos 2021, Tsonaka and Spitali 2021). Other software packages, such as mgcv (Wood 2011), brms (Bürkner 2017), gamlss (Rigby and Stasinopoulos 2005), and NBZIMM (Zhang and Yi 2020) can also be used to analyze overdispersed or sparse count data. These packages are not specifically designed for RNA-seq data analysis.

To address these challenges, hierarchical mixed-effects models based on the negative-binomial (NB) distribution can be considered (Molenberghs *et al.* 2010). The suitability of the models for the analysis of clustered and longitudinal RNA-seq data was confirmed by extensive simulations and analyses of real-life data by Kazakiewicz *et al.* (2019) and Tsonaka and Spitali (2021).

Given the suitability of the models, an important question is the availability and reliability of software that would allow applying the models in practice. It appears that this type of models has been implemented in commercial software such as **SAS** and **STATA**, as well as in open-source platforms such as **R**. In **R**, there are multiple packages that could be used for fitting these models, including lme4 (Bates *et al.* 2015), GLMMadaptive (Rizopoulos 2022), glmmTMB (Brooks *et al.* 2017), glmmADMB (Skaug *et al.* 2016). However, all these implementations differ in terms of the complexity of the random-effects structure that can be specified, optimization methods, and computational speed.

Thus, to help researchers in choosing from the available tools, we compare, in this paper, several of the implementations and evaluate their suitability for applying the hierarchical NB model with normally distributed random effects to account for the within- and between-sample correlation (Kazakiewicz *et al.* 2019). Toward this aim, we use real-life data from an RNA-seq experiment.

The paper is structured as follows. Section 2 describes the hierarchical NB model (Section 2.1), its estimation (Section 2.2), and software implementations (Section 2.3). Section 2.4 provides a description of the real-life RNA-seq data that are used to illustrate the application of the model and various software. Sections 3 present the results of the application. A short discussion in Section 4 concludes the paper.

## 2 Methods

In this section, we provide a description of the hierarchical NB model, its estimation, and various software implementations.

### 2.1 The model

We consider per-gene analysis. Thus, in what follows, we drop the index indicating the gene.

Assume that, for a particular gene, a column vector of read counts (corresponding to, for instance, multiple exons), $y_{s}={\left(y_{s1},\ldots ,y_{sJ}\right)}^{\prime }$, is available for sample *s*. Additionally, the sample is described by a vector of variables $x_{s}={\left(1,x_{s1},\ldots ,x_{sp}\right)}^{\prime }$.

To account for overdispersion and possible correlation between the counts, we consider the following two-level (with counts grouped within samples) hierarchical model:


(1) $$y_{sj}|\gamma_{s},b_{s}∼\text{Poisson}(T_{sj}\gamma_{s}e^{x_{s}^{\prime }\beta +b_{s}}),$$

(2) $$\gamma_{s}∼\text{Gamma}(\phi ,1/\phi ),$$

(3) $$b_{s}∼\text{Normal}(0,\sigma^{2}),$$

where $\beta ={\left(\beta_{0},\beta_{1},\ldots ,\beta_{p}\right)}^{\prime }$ is the vector of coefficients that captures the effect of variables $x_{s}$, $\gamma_{s}$ is a gamma-distributed random effect with an overdispersion parameter ϕ, $b_{s}$ is a normally distributed random effect, and $T_{sj}$ is an “exposure” (“normalization”) factor related to, for instance, the library size (a total number of read counts for sample *s*). Note that other normalization factors can be considered, such as the factors obtained by the Trimmed Mean of M-values normalization implemented in edgeR (Robinson *et al.* 2010) or by the “median of ratios” normalization found in DESeq2 (Love *et al.* 2014). The model implies that, conditionally on $b_{s}$, $y_{s}$ is distributed according to a multivariate NB (MVNB) distribution (Fabio *et al.* 2012, Kazakiewicz *et al.* 2019) with the probability mass function given by:


(4) $$P(y_{s}|b_{s})=\frac{\Gamma (\phi +\sum_{j=1}^{J}y_{sj})}{\Gamma (\phi )\Gamma (\sum_{j=1}^{J}y_{sj!})}Q_{s}^{-\phi }\prod_{j=1}^{J}\{(\phi Q_{s}{)}^{-1}T_{sj}e^{x_{s}^{\prime }\beta +b_{s}}{\}}^{y_{sj}},$$

where


(5) $$Q_{s}=1+\phi^{-1}\sum_{j=1}^{J}T_{sj}e^{x_{s}^{\prime }\beta +b_{s}}.$$

The marginal mean and variance of the exon count $y_{sj}$ are given by, respectively,


(6) $$E(y_{sj})=T_{sj}e^{x_{s}^{\prime }\beta }e^{\sigma^{2}/2},$$

(7) $$\text{Var}(y_{sj})=E(y_{sj})\left\{1+e^{\sigma^{2}}\left(1/\phi +1-e^{-\sigma^{2}}\right)E(y_{sj})\right\}.$$

As the marginal variance is larger than the mean value, the model implies overdispersion, as compared to the Poisson distribution.

The marginal correlation between the *j*-th and *k*-th count observed for the same sample *s* is given by:


(8) $$\text{Cor}(y_{sj},y_{sk})=\frac{E(y_{sj})E(y_{sk})e^{\sigma^{2}}\left(1/\phi +1-e^{-\sigma^{2}}\right)}{\sqrt{\text{Var}(y_{sj})\text{Var}(y_{sk})}}.$$

The marginal likelihood corresponding to models (1–3) is given by:


(9) $$L(\beta ,\phi ,\sigma^{2})=\int_{-\infty }^{\infty }\prod_{s=1}^{S}P(y_{s}|b_{s})\varphi (b_{s};\sigma^{2})db_{s},$$

where $\varphi (x;\sigma^{2})$ denotes the PDF of a mean-zero normal distribution with variance $\sigma^{2}$. In general, the integral in (9) does not have an analytical expression, but it can be computed numerically.

The model can be extended to accommodate more complex designs. For instance, read counts may be obtained for multiple samples derived for the same individual or cluster. As a result, there may be a correlation between the counts obtained in different samples. In this case, denote by $y_{\mathrm{isj}}$ the *j*-th count obtained for the *s*-th sample ($s=1,\ldots ,n_{i}$) for individual/cluster *i* (i=1,…,N). Let $y_{is}={\left(y_{is1},\ldots ,y_{\mathrm{isJ}}\right)}^{\prime }$ be the column vector collecting all the *J* counts. Additionally, let $x_{is}={\left(1,x_{is1},\ldots ,x_{\mathrm{isp}}\right)}^{\prime }$ be the column vector of variables describing sample *s* for individual/cluster *i*. We can then consider the following three-level hierarchical model:


(10) $$y_{\mathrm{isj}}|\gamma_{is},b_{i},b_{is}∼\text{Poisson}(T_{\mathrm{isj}}\gamma_{is}e^{x_{is}^{\prime }\beta +b_{i}+b_{is}}),$$

(11) $$\gamma_{is}∼\text{Gamma}(\phi ,1/\phi ),$$

(12) $$b_{i}∼\text{Normal}(0,\sigma_{I}^{2}),$$

(13) $$b_{is}∼\text{Normal}(0,\sigma_{S}^{2}).$$

The random effects $b_{i}$ are independent of $b_{is}$ and imply correlation between counts for different samples from the same cluster/individual, while effects $b_{s}$ allow for the within-sample correlation of the counts. The marginal likelihood for models (10–13) is obtained as for models (1–3), i.e., by integrating the conditional MVNB distribution over the joint distribution of $b_{i}$ and $b_{is}$.

The marginal mean and variance of the exon count $y_{\mathrm{isj}}$ are given by, respectively,


(14) $$E(y_{\mathrm{isj}})=T_{\mathrm{isj}}e^{x_{is}^{\prime }\beta }e^{(\sigma_{I}^{2}+\sigma_{S}^{2})/2},$$

(15) $$\text{Var}(y_{\mathrm{isj}})=E(y_{\mathrm{isj}})\left\{1+e^{\sigma_{I}^{2}+\sigma_{S}^{2}}\left(1/\phi +1-e^{-\sigma_{I}^{2}-\sigma_{S}^{2}}\right)E(y_{\mathrm{isj}})\right\}.$$

The marginal correlation between the *j*-th and *k*-th count observed for the same sample *s* of individual *i* is given by:


(16) $$\text{Cor}(y_{\mathrm{isj}},y_{\mathrm{isk}})=\frac{E(y_{\mathrm{isj}})E(y_{\mathrm{isk}})e^{\sigma_{I}^{2}+\sigma_{S}^{2}}\left(1/\phi +1-e^{-\sigma_{I}^{2}-\sigma_{S}^{2}}\right)}{\sqrt{\text{Var}(y_{\mathrm{isj}})\text{Var}(y_{\mathrm{isk}})}}.$$

On the other hand, the correlation between the *j*-th and *k*-th count observed for different samples *s* and *t* of individual *i* is given by:


(17) $$\text{Cor}(y_{\mathrm{isj}},y_{\mathrm{itk}})=\frac{E(y_{\mathrm{isj}})E(y_{\mathrm{itk}})\left(e^{\sigma_{I}^{2}}-1\right)}{\sqrt{\text{Var}(y_{\mathrm{isj}})\text{Var}(y_{\mathrm{itk}})}}.$$

### 2.2 Estimation

Coefficients of models (1–3) or (10–13) may be estimated by maximizing the marginal likelihood. As mentioned in Section 2.1, the likelihood is obtained by integrating out the random effects and does not have an analytical expression. Thus, the likelihood has to be computed numerically. Toward this aim, various approaches can be applied. The two most often applied techniques, on which we will focus, are the Laplace approximation and the adaptive Gauss-Hermite quadrature (AGHQ).

The Laplace approximation uses an approximation of the likelihood by a Gaussian (normal) distribution around its maximum. This makes the log-likelihood function quadratic and allows for the use of a second-order Taylor expansion (Vonesh 1996, Bolker *et al.* 2009).

The AGHQ is a numerical algorithm used to approximate integrals. It improves the standard Gauss-Hermite quadrature by dynamically adjusting nodes and weights to more accurately approximate integrals (Pinheiro and Chao 2006). The AGHQ is more accurate than the Laplace approximation. However, as the number of random effects and/or quadrature points increases, the AGHQ’s computation time also increases. As a result, the AGHQ is not feasible for models that involve more than two or three random factors (Bolker *et al.* 2009). It is worth noting that the AGHQ with only a single quadrature point corresponds to the Laplace approximation.

### 2.3 Software

As it has been mentioned in Section 1, hierarchical models based on the NB distribution have been implemented in several commercial and open-source software platforms. In what follows, we consider the implementation in commercial software **SAS** (procedure NLMIXED, [Inc. 2023]) and **Stata** (command menbreg, [StataCorp. LLC 2023]). Additionally, we include four packages (GLMMadaptive, lme4, glmmTMB, and glmmADMB), available in **R** (Bates *et al.* 2015, Brooks *et al.* 2017, Rizopoulos 2022). Several of the implementations (GLMMadaptive, lme4, NLMIXED, menbreg) apply the AGHQ that is known to be preferred from a numerical-accuracy point of view. GLMMadaptive allows only estimation of two-levels model, i.e., models with random effects corresponding to only one grouping factor; the other implementations can be used to fit models with more complex random-effect structures. Table S1, available as supplementary data at *Bioinformatics Advances* online, provides a summary of the considered implementations. In what follows, we briefly describe each of the implementations.

#### 2.3.1 SAS

Procedure NLMIXED in **SAS** (SAS Institute Inc. 2023) allows specifying a conditional distribution of data given normally distributed random effects. The resulting model is estimated by maximizing (an approximation of) the marginal likelihood obtained by integration of the conditional likelihood over the distribution of the random effects. The procedure allows using the AGHQ to approximate the marginal likelihood. Multi-level models with random effects for several (nested) grouping factors can be considered. The use of the NLMIXED procedure to analyze overdispersed count data was described, for instance, by (Molenberghs *et al.* 2007). In the remainder of the paper, we will refer to this procedure as “SAS.”

#### 2.3.2 STATA

Command menbreg in **STATA** (StataCorp. LLC 2023) allows estimation of multilevel mixed-effects NB regression models to count data. Models with random effects for several (nested or crossed) grouping factors can be considered. The estimation is based on maximizing (an approximation of) the marginal likelihood. The likelihood may be approximated by using the Laplace approximation or the AGHQ. In the remainder of the paper, we will refer to the use of menbreg command with the AGHQ as “STATA.”

#### 2.3.3 lme4

The **lme4** package (Bates *et al.* 2015) in **R** provides functions to fit and analyze a generalized linear mixed-effects model (GLMM). Specifically, the **glmer.nb** function can be used to fit a GLMM with an NB-distributed response, designed to handle count data that exhibit overdispersion. The function incorporates both fixed and random effects in a linear predictor. The dispersion parameter is automatically estimated during the model fitting process. The **lme4** package supports complex structures of the fixed and random effects using a simple syntax. Additionally, two methods for approximating the likelihood are implemented. The nAGQ argument (from the **glmer.nb** function) controls the number of nodes in the quadrature formula, and thus the approximation method. Setting nAGQ equal to 1 corresponds to the Laplace approximation, while values greater than 1 indicate the number of points used to evaluate the AGHQ approximation to the log-likelihood. In the rest of the paper, we will refer to these approaches as “LME4L” and “LME4A,” respectively.

#### 2.3.4 glmmTMB

The **glmmTMB** package (Brooks *et al.* 2017) in **R** allows fitting complex GLMMs for a wide range of response distributions. The models are fitted via Template Model Builder (TMB), which maximizes flexibility and speed. **glmmTMB** supports complex random effects structures with multiple levels of nested or crossed random effects. The package uses the same formula syntax as **lme4**, and the Laplace approximation to compute the marginal likelihood. In the rest of the paper, we will refer to this method as “TMB.”

#### 2.3.5 glmmADMB

The **glmmADMB** package (Fournier *et al.* 2012) in **R** applies automatic differentiation to fit non-linear models with a large number of parameters (Skaug *et al.* 2016). Similarly to **glmmTMB**, **glmmADMB** supports a wide variety of response distributions, link functions, and complex random effects structures. The package uses the Laplace approximation to compute the marginal likelihood. For the remainder of this paper, we will refer to this method as “ADMB.”

#### 2.3.6 GLMMadaptive

The **GLMMadaptive** package (Rizopoulos 2022) in **R** allows fitting GLMMs for grouped or clustered responses for which the marginal likelihood is approximated by using the AGHQ. Although multiple random effects (e.g. random intercepts, linear and quadratic random slopes) are allowed, there is no possibility to include nested or crossed random effects designs. **GLMMadaptive** has a slightly different formula syntax as compared to **lme4**. The formulas for the fixed and random effects are specified in separate arguments of the function. In the rest of the paper, we will refer to this method as “GLMMa.”

### 2.4 Data

We illustrate the use of different implementations of the hierarchical NB model by using a dataset obtained from an RNA-seq experiment (Bouquet *et al.* 2016).

The dataset includes samples from 29 patients with tick-borne Lyme disease and 13 healthy controls. Patients with the disease were enrolled at the time of diagnosis and followed for up to 6 months (Bouquet *et al.* 2016). From each patient, blood samples were taken at three time points after infection: before the antibiotic treatment (visit1), immediately after the treatment (visit2), and 6 months after treatment completion (visit3). Samples for healthy controls were obtained at one time point. Three samples for patients were discarded because of insufficient read counts and transcriptome coverage. Data were processed, including quality control, read trimming, alignment, and quantification. As a result, 137 078 exons with a nonzero sum of counts across all samples, grouped into 18 765 protein-coding genes, were included in the analysis. The raw data are publicly available under GEO accession number GSE63085.

We consider per-gene analysis. It is worth noting that, for a particular gene, counts obtained for different exons in the same sample may be correlated. Additionally, for patients, there may be correlation between counts obtained for the same patient in different samples. To reflect the correlation structure, which results from the hierarchical structure of the data, we may assume that, for a particular gene, the exon count corresponding to exon *j* from sample *s* for individual *i* follows the three-level model (10–13), with the conditional Poisson distribution expressed as follows:


(18) $$\begin{array}{c} y_{\mathrm{isj}}|\gamma_{is},b_{i},b_{is}∼\\ \text{Poisson}(T_{\mathrm{isj}}\gamma_{is}e^{\beta_{0}+\beta_{1}\;\text{visit}1+\beta_{2}\text{visit}2+\beta_{3}\text{visit}3+b_{i}+b_{is}}), \end{array}$$

where $\beta_{1}$, $\beta_{2}$, and $\beta_{3}$ are the coefficients capturing the relative change of the average gene-level expression intensity for patients at visit1, visit2, and visit3, respectively, as compared to the healthy controls. The “exposure” $T_{\mathrm{isj}}$ is defined as $d_{j}\times l_{is}$, where $d_{j}$ is the exon length and $l_{is}$ it the total library size (Kazakiewicz *et al.* 2019).

Apart from the estimates of the coefficients of the model, we are interested in testing the following three null hypotheses:

-
$H_{0}^{123}:\beta_{1}=\beta_{2}=\beta_{3}=0$

-
$H_{0}^{1}:\beta_{1}=0$

-
$H_{0}^{3}:\beta_{3}=0$

Rejection of $H_{0}^{123}$ implies that gene expression for patients, as compared to healthy controls, is different at least at one of the three time points. Conducting this test might be of interest when, for instance, screening for genes that may be related to the mechanism of the Lyme disease or its treatment. Rejection of $H_{0}^{1}$ means that gene expression for patients is, on average, different as compared of healthy individuals. Testing this hypothesis might be of interest in screening for genes that may be related to the mechanism of the Lyme disease. Finally, rejection of $H_{0}^{3}$ implies that, despite treatment, there may be a difference in gene expression between the two groups. Testing this hypothesis might be of interest when screening for genes that may be useful, for instance, for explaining the mechanism of treatment.

The null hypotheses can be tested by using the chi-squared Wald-test statistics with 3 ($H_{0}^{123}$) or 1 ($H_{0}^{1}$ and $H_{0}^{3}$) degrees of freedom (Galecki and Burzykowski 2013).

Fitting the three-level model (18) to the data may be computationally difficult because it includes normally distributed random effects at two levels (individual and sample). Also, as mentioned in Section 2.3, not all software implementations allow fitting such a model. Thus, as an alternative, we may consider a simpler, two-level model without the individual-specific random effects $b_{i}$:


(19) $$\begin{array}{c} y_{\mathrm{isj}}|\gamma_{is},b_{is}∼\\ \text{Poisson}(T_{\mathrm{isj}}\gamma_{is}e^{\beta_{0}+\beta_{1}\;\text{visit}1+\beta_{2}\text{visit}2+\beta_{3}\text{visit}3+b_{is}}). \end{array}$$

One could expect that the related variability would be additionally captured by the sample-specific random effects $b_{is}$.

Because of computational difficulties, we will compare the results of fitting the three-level model (18) by using different software implementations only for one exemplary gene. However, the results for the two-level model (19) will be compared for all genes.

## 3 Results

In Section 3.1, we present the results of fitting the three-level model (18) and the simpler two-level model (19) to data for a single gene from the RNA-Seq dataset by using the different software implementations mentioned in Section 2.3. Additionally, in Section 3.2, we compare the results of fitting the simpler model for all genes.

### 3.1 Analysis of data for a single gene

For the selected gene, ENSG00000144802, the data contains read counts from 11 exons. The exon length ranges from 76 (exon ENSE00001517391) to 1705 (exon ENSE00000967213) nucleotides (see Table 1). The exons ENSE00000967205, ENSE00000967213, and ENSE00001248707 exhibited the highest coverage per base pair, with an average coverage ranging from 2 to 8, and from 1 to 2 reads per base pair for the patients and controls, respectively.

**Table 1.** Exon-specific sample means and variances for three control samples with library size about $1.7\times 10^{6}$ and for three control samples with library size about $3.5\times 10^{6}$.^a^

Exon	Exon length	Library size			
		$∼1.7\times 10^{6}$		$∼3.5\times 10^{6}$	
		Mean	Variance	Mean	Variance
ENSE00000967205	104	71.7	2344	119.7	2497
ENSE00000967207	106	6.3	12.3	20	57
ENSE00000967213	1705	2098	5316012	3833	2524851
ENSE00001248707	773	594.3	410006	1217	242954
ENSE00001517391	76	10	63	33.3	732.3
ENSE00001822656	503	163.7	27414	474.7	17170
ENSE00001858993	1026	3.7	40.3	6	31
ENSE00001936731	81	3	13	8.7	2.3
ENSE00001939990	623	42.7	676.3	98.7	576.3
ENSE00001946670	164	58.7	2736	123.7	2116
ENSE00001958418	260	4.3	20.3	4.3	5.3

a Gene ENSG00000144802.

**Figure 1.**
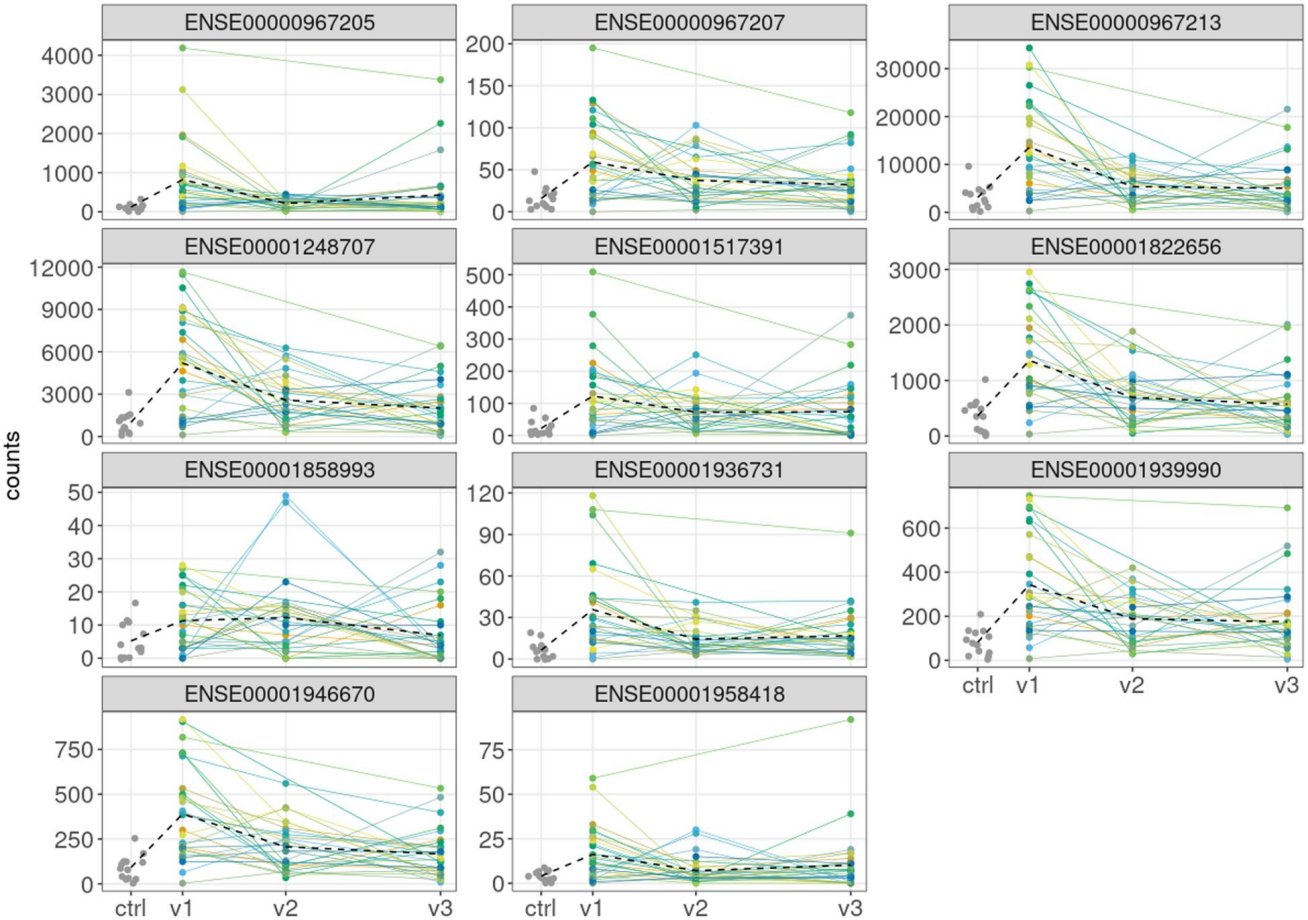
Exon counts for gene ENSG00000144802. The grey points at the left-hand side of each plot indicate the exon counts for 13 healthy controls. The colored lines represent the profiles of 29 patients across three visits, labeled as v1, v2, and v3. The black dashed line links the mean of the control counts and the visit-specific means for patients. Note that different plots use different y-scales.

Figure 1 presents the observed counts per exon for controls and the three visits for patients. The black dashed line links the mean count for the control group with the visit-specific means for the patients. For each exon, an increase in the average expression level at the first (pre-treatment visit) is observed for patients as compared to the control group. For patients, a descending average profile can be seen for six exons (ENSE00000967207, ENSE00000967213, ENSE00001248707, ENSE00001822656, ENSE00001939990, ENSE00001946670). In contrast, four exons (ENSE00000967205, ENSE00001517391, ENSE00001936731, ENSE00001958418) show a decrease from visit1 to visit2, followed by a slight increase from visit2 to visit3. For exon ENSE00001858993, a slight increase is observed between visit1 and visit2.

For most exons, Fig. 1 illustrates a considerable variability of counts for the control group and for the patients. In part, this variability is due to varying library sizes for the samples, for which the counts were obtained. However, even after taking into account the library size, excess variability, as compared to the Poisson distribution, is detected. This is illustrated in Table 1. The table presents, for each exon, the sample mean and variance of the counts for three control samples with the library size about $1.7\times 10^{6}$ reads and for three control samples with the library size about $3.5\times 10^{6}$ reads. In all but one case, the mean is smaller than the variance and indicates overdispersion. This justifies the use of the NB distribution for the analysis of the counts.

Table 2 and Table S2, available as supplementary data at *Bioinformatics Advances* online, show the estimates of the coefficients of the three-level model (18) and the computational time for various software. Default starting values and convergence settings were used; the syntax used to fit the model is provided on Zenodo (https://doi.org/10.5281/zenodo.14908112). All analyses for the gene were performed on a machine with an i7-11850H processor @2.50 GHz, 2496 MHz, 8 cores, and 32.0 GB of installed physical memory. Note that LME4A and GLMMa were not included in this comparison, as they only allow fitting two-level models. For the implementations based on the AGHQ approximation, 75 quadrature points were used.

**Table 2.** Estimates of the coefficients and computational time for the three-level model (18), see Section 2.4.^a^

	Estimates							
Method	$\beta_{0}$	$\beta_{1}$	$\beta_{2}$	$\beta_{3}$	$\sigma_{S}$	$\sigma_{I}$	ϕ	Time (s)
LME4L	−15.1390	0.7230	0.5330	0.1478	0.5062	0.2537	26429.5300	7.78
TMB	−15.4359	0.7495	0.5151	0.3254	0.2262	0.2297	0.5112	1.97
ADMB	−15.4359	0.7495	0.5151	0.3254	0.2262	0.2297	0.5112	71.42
STATA	−15.4384	0.7496	0.5154	0.3257	0.2236	0.2321	0.5112	308.46
SAS	−15.4360	0.7520	0.5178	0.3281	0.2234	0.2328	0.5112	378.90

a Gene ENSG00000144802. LME4L—**lme4** with Laplace approximation; TMB—**glmmTMB**; ADMB—**glmmadmb**; SAS—**PROC NLMIXED**; STATA—**menbreg**. For the AGHQ-based methods, 75 quadrature points were used.

The estimates of the coefficients of the model are consistent across the different software, except of LME4L. For the latter implementation, especially striking is the overestimation of the variance-structure coefficients $\sigma_{I}$ (individual-level random-effect standard deviation), $\sigma_{S}$ (sample-level random-effect standard deviation), and ϕ (dispersion parameter).

In terms of computational time, there is a clear difference between the implementations that used the Laplace approximation (LME4L, TMB, ADMB) and the AGHQ (SAS, STATA) for the numerical computation of the marginal likelihood. Among the four implementations that yielded similar results, TMB was the fastest, while SAS was the slowest.

Additionally, model (18) was fitted by SAS and STATA while using varying numbers (5, 10, 25, 50, and 75) of quadrature points in the AGHQ approximation. Table 3 presents the obtained estimates and computational time. Clearly, increasing the number of quadrature points results in a longer computational time, though the STATA implementation is consistently faster than the SAS one. The table suggests that, starting from 10 quadrature points, there is little to no difference in the obtained estimates. Thus, increasing the number of quadrature points beyond 10 added computational complexity without substantial improvement in accuracy.

**Table 3.** Estimates of the coefficients of the three-level model (18) fitted by using different numbers of quadrature points (column AGHQ).^a^

		Estimates							
AGHQ	Method	$\beta_{0}$	$\beta_{1}$	$\beta_{2}$	$\beta_{3}$	$\sigma_{S}$	$\sigma_{I}$	ϕ	Time (s)
*n* = 5	SAS	−15.4389	0.7525	0.5183	0.3285	0.2233	0.2330	0.5112	30.54
	STATA	−15.4360	0.7496	0.5154	0.3257	0.2236	0.2321	0.5112	0.29
*n* = 10	SAS	−15.4384	0.7520	0.5178	0.3280	0.2234	0.2328	0.5112	35.97
	STATA	−15.4360	0.7496	0.5154	0.3257	0.2236	0.2321	0.5112	0.79
*n* = 25	SAS	−15.4384	0.7520	0.5178	0.3281	0.2234	0.2328	0.5112	70.31
	STATA	−15.4360	0.7496	0.5154	0.3257	0.2236	0.2321	0.5112	4.24
*n* = 50	SAS	−15.4384	0.7520	0.5178	0.3281	0.2234	0.2328	0.5112	195.55
	STATA	−15.4360	0.7496	0.5154	0.3257	0.2236	0.2321	0.5112	140.85
*n* = 75	SAS	−15.4384	0.7520	0.5178	0.3281	0.2234	0.2328	0.5112	378.90
	STATA	−15.4360	0.7496	0.5154	0.3257	0.2236	0.2321	0.5112	308.46

a Gene ENSG00000144802. SAS—**PROC NLMIXED**; STATA—**menbreg**.

Based on the results presented in Table 2 it is possible to investigate the correlation between the counts. For instance, consider exons ENSE00000967205 and ENSE00000967207 of length 104 and 106, respectively (see Table 1), and two control samples with library sizes of $1.7\times 10^{6}$ and $3.5\times 10^{6}$. By using formula (16) and estimates provided in Table 2 for STATA, we can compute that the correlation coefficient for the counts for the two exons in the same control sample with library size of $1.7\times 10^{6}$ is equal to 0.988, while in a control sample with size $3.5\times 10^{6}$ it is equal to 0.994. On the other hand, by using formula (17), the correlation coefficient for a count for exon ENSE00000967205 in a control sample with library size of $1.7\times 10^{6}$ and a count for exon ENSE00000967207 in a different sample (but from the same control individual) with library size of $3.5\times 10^{6}$ is equal to 0.024. Thus, while the within-sample correlation between the exon counts is considerable, the between-sample correlation is negligible. This might be taken as an argument in favor of using the simpler, two-level model (19), because the model does not include individual-specific random effects $b_{i}$. Consequently, it assumes that there is no between-sample correlation between exon counts.

The simpler, two-level model (19) was fitted to the data by using seven different implementations, including LME4A and GLMMa. Default starting values and convergence settings were used. For the implementations based on the AGHQ approximation, 75 quadrature points were used. Table 4 shows the parameter estimates. As in the case (see Table 2) of the three-level model, estimates of the coefficients of model (19) are consistent across the different implementations, except of LME4L. The AGHQ-based implementations are slower than the Laplace-approximation-based ones, but for all implementations, computations require less time than for the three-level model (18).

**Table 4.** Estimates of the coefficients and computational time for the two-level model (19), see Section 2.4.^a^

	Estimates						
Method	$\beta_{0}$	$\beta_{1}$	$\beta_{2}$	$\beta_{3}$	σ	ϕ	Time (s)
LME4L	−15.1390	0.7164	0.5194	0.1479	0.5657	26429.5300	6.85
LME4A	−15.1390	0.7164	0.5194	0.1479	0.5657	26429.5300	50.93
GLMMa	−15.4351	0.7471	0.4987	0.3246	0.3156	0.5107	1.19
TMB	−15.4351	0.7469	0.4988	0.3242	0.3169	0.5108	1.64
ADMB	−15.4350	0.7469	0.4988	0.3242	0.3169	0.5108	20.02
STATA	−15.4349	0.7468	0.4985	0.3245	0.3154	0.5108	2.29
SAS	−15.4349	0.7468	0.4985	0.3245	0.3154	0.5108	7.17

a Gene ENSG00000144802. LME4L—**lme4** with the Laplace approximation; LME4A—**lme4** with the adaptive Gaussian quadrature; GLMMa—**GLMMadaptive**; TMB—**glmmTMB**; ADMB—**glmmadmb**; SAS—**PROC NLMIXED**; STATA—**menbreg**. For the AGHQ-based methods, 75 quadrature points were used.

Table 5 presents the estimates and the computational time for the two-level model (19) obtained while using varying numbers (5, 10, 25, 50, and 75) of quadrature points in the AGHQ approximation. Across all methods, except of LME4A, the estimates are quite consistent when the number of quadrature points is at least 10. The computational time clearly increases with the increasing number of the points. In terms of the time, STATA seems to be comparable to GLMMa, while they both are faster than SAS.

**Table 5.** Estimates of the coefficients of the two-level model (19) obtained by using different number of quadrature points (column AGHQ).^a^

		Estimates						
AGHQ	Method	$\beta_{0}$	$\beta_{1}$	$\beta_{2}$	$\beta_{3}$	σ	ϕ	Time (s)
***n* = 5**	LME4A	−15.1390	0.7164	0.5194	0.1479	0.5657	26429.5300	7.13
	GLMMa	−15.7868	0.9534	0.5941	0.4217	0.4955	0.5317	0.47
	SAS	−15.4349	0.7468	0.4985	0.3245	0.3154	0.5108	0.81
	STATA	−15.4349	0.7468	0.4985	0.3245	0.3155	0.5108	0.09
***n* = 10**	LME4A	−15.1390	0.7164	0.5194	0.1479	0.5657	26429.5300	8.32
	GLMMa	−15.4351	0.7468	0.4988	0.3243	0.3165	0.5109	0.68
	SAS	−15.4349	0.7468	0.4985	0.3245	0.3154	0.5108	1.27
	STATA	−15.4349	0.7468	0.4985	0.3245	0.3155	0.5108	0.10
***n* = 25**	LME4A	−15.1390	0.7164	0.5194	0.1479	0.5657	26429.5300	12.26
	GLMMa	−15.4350	0.7460	0.4989	0.3214	0.3237	0.5112	0.81
	SAS	−15.4349	0.7468	0.4985	0.3245	0.3154	0.5108	2.87
	STATA	−15.4349	0.7468	0.4985	0.3245	0.3155	0.5108	0.15
***n* = 50**	LME4A	−15.1390	0.7164	0.5194	0.1479	0.5657	26429.5300	19.18
	GLMMa	−15.4351	0.7471	0.4987	0.3246	0.3156	0.5107	1.10
	SAS	−15.4349	0.7468	0.4985	0.3245	0.3154	0.5108	5.04
	STATA	−15.4349	0.7468	0.4985	0.3245	0.3155	0.5108	1.55
***n* = 75**	LME4A	−15.1390	0.7164	0.5194	0.1479	0.5657	26429.5300	32.57
	GLMMa	−15.4351	0.7471	0.4987	0.3246	0.3156	0.5107	1.76
	SAS	−15.4349	0.7468	0.4985	0.3245	0.3154	0.5108	7.17
	STATA	−15.4349	0.7468	0.4985	0.3245	0.3155	0.5108	2.29

a Gene ENSG00000144802. LME4A—**lme4** with the AGHQ approximation; GLMMa—**GLMMadaptive**; SAS—**PROC NLMIXED**; STATA—**menbreg**.

It is worth noting that, for all the implementations except LME4, the estimated values of coefficients $\beta_{1}$, $\beta_{2}$, and $\beta_{3}$ from Table 4 are very close to their counterparts from Table 2. Thus, both models provide similar conclusions regarding the average difference between the patients’ and control samples.

Additionally, it is worth observing that, for all the implementations except LME4, the estimated value of the between-sample variance $\sigma^{2}$ of model (19) is close to the sum of the between-individual variance $\sigma_{I}^{2}$ and the between-sample variance $\sigma_{S}^{2}$ from the three-level model (18). For instance, for STATA (see Table 2), $\sigma_{I}^{2}+\sigma_{S}^{2}=0.2321^{2}+0.2236^{2}=0.1039$ for (18), which is close (see Table 4) to $\sigma^{2}=0.3154^{2}=0.0995$ obtained for model (19). This implies, upon noting that Eqs. (6–8) and (14–16) are identical if $\sigma^{2}=\sigma_{I}^{2}+\sigma_{S}^{2}$, that the within-sample correlation estimated for the two-level model (19) should correspond to the estimates obtained for three-level model (18). This is indeed the case: by applying Eq. (8) to the results presented in Table 4 for STATA, we can compute that the correlation coefficient for the counts for exons ENSE00000967205 and ENSE00000967207 in a control sample with library size of $1.7\times 10^{6}$ is equal to 0.988, while in a control sample with size $3.5\times 10^{6}$ it is equal to 0.994. These results are identical to those reported for Table 2. Thus, the simpler model (19) provides the same conclusion regarding the correlation structure as the more complex model (18): a substantial within-sample correlation between exon counts and a negligible between-sample correlation.

In the next section, we present results of the estimation of the two-level model (19) for all genes and various software implementations. Note that glmmADMB was used only for the analysis of a single gene. It has been replaced by glmmTMB, which is optimized for speed and memory efficiency and is actively maintained, unlike glmmADMB.

### 3.2 Analysis of data for all genes

The simpler, two-level model (19) was fitted for 18 765 genes. Default starting values and convergence settings were used. For the implementations based on the AGHQ approximation, 75 quadrature points were used. Table S3, available as supplementary data at *Bioinformatics Advances* online, summarizes the number of genes for which the model was successfully fitted. The model was considered to fail due to either non-convergence, non-estimable variance-covariance matrix of the coefficients, or the matrix not being positive definite. The number of genes for which the model could be successfully fitted is the largest for TMB, followed by GLMMa, SAS, and STATA. For 9112 genes, the model could be fitted by all the software implementations.

**Figure 2.**
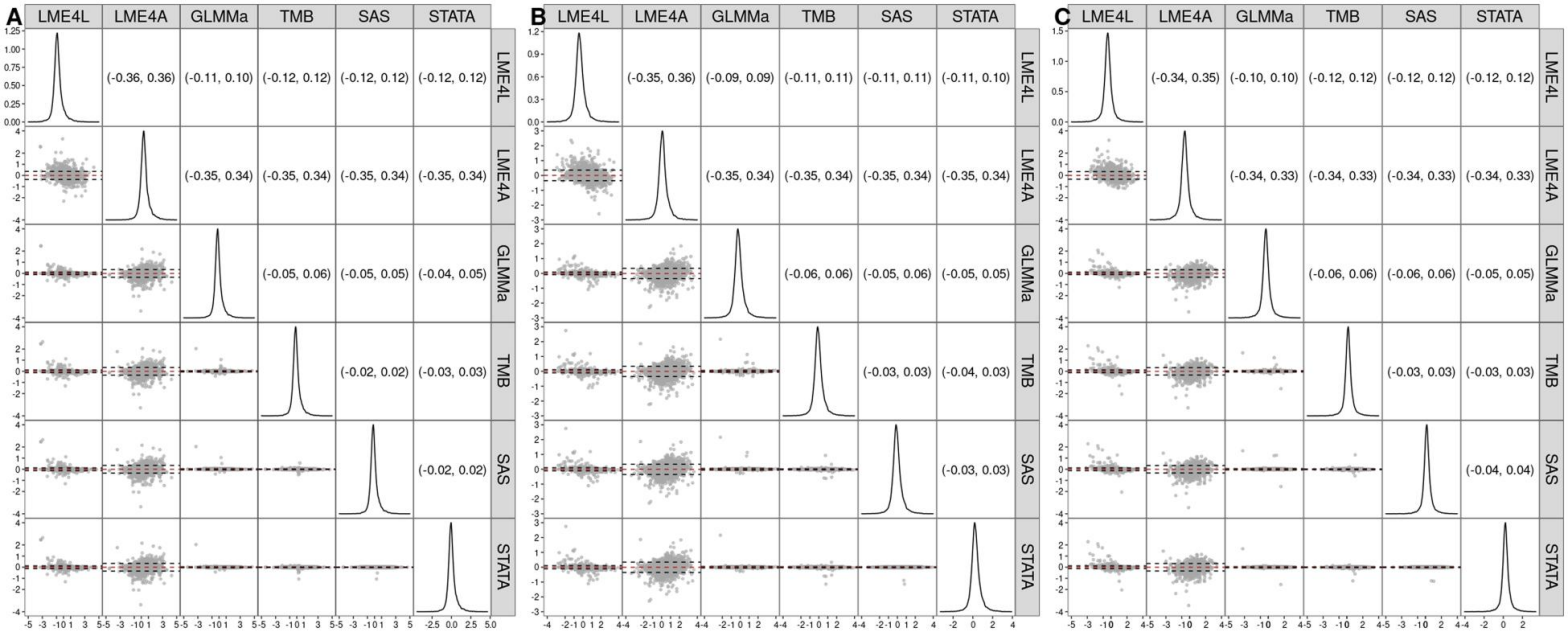
Estimates of the mean-structure coefficients of the two-level model (19) for the different software implementations and the 9112 genes, for which the model was successfully fitted for all the implementations. The densities of the estimates are shown along the diagonal. The lower triangle contains Bland–Altman plots of the estimates for pairs of different software implementations, with the red dashed line indicating the mean difference and the black dashed lines marking the 95% limits of agreement. The upper triangle presents the numerical values of the limits. Panel A: $\beta_{1}$; Panel B: $\beta_{2}$; Panel C: $\beta_{3}$. LME4L—**lme4** with the Laplace approximation; LME4A—**lme4** with the AGHQ approximation; GLMMa—**GLMMadaptive**; TMB—**glmmTMB**; SAS—**PROC NLMIXED**; STATA—**menbreg**.

Figure 2 shows a scatterplot matrix that contains (under the diagonal) the Bland–Altman plots (Bland and Altman 1999) for the estimates of the mean-structure coefficients ($\beta_{1}$ in Panel A, $\beta_{2}$ in Panel B, and $\beta_{3}$ in Panel 3) for the different implementations and the 9112 genes, for which the two-level model (19) was successfully fitted for all the implementations. In particular, the scatterplots present, for each pair of the implementations, the difference in the estimates (on the y-axis) as a function of the average of the estimates (on the x-axis). The red dashed line indicates the mean difference; ideally, the mean should be equal to 0, because this implies no difference (on average) between the estimates obtained by two implementations. Additionally, the black dashed lines in the plots mark the 95% limits of agreement (constructed as mean±1.96×SD, where SD is the standard deviation of the differences). The limits are also presented numerically above the diagonal in each panel. They can be interpreted as the region that should contain 95% of the differences between estimates for a pair of implementations. Thus, the narrower the limits, the better agreement between the estimates obtained by two implementations.

The Bland-Altman plots in all three panels of Fig. 2 indicate that the mean difference is close to 0 (see also Table S4, available as supplementary data at *Bioinformatics Advances* online). The 95% limits of agreement are the widest for LME4A (around 0.7). On the other hand, they are much more narrow (with the width ranging from 0.05 to 0.12) for GLMMa, TMB, SAS, and STATA, suggesting that these four implementations yield very similar estimates.

**Figure 3.**
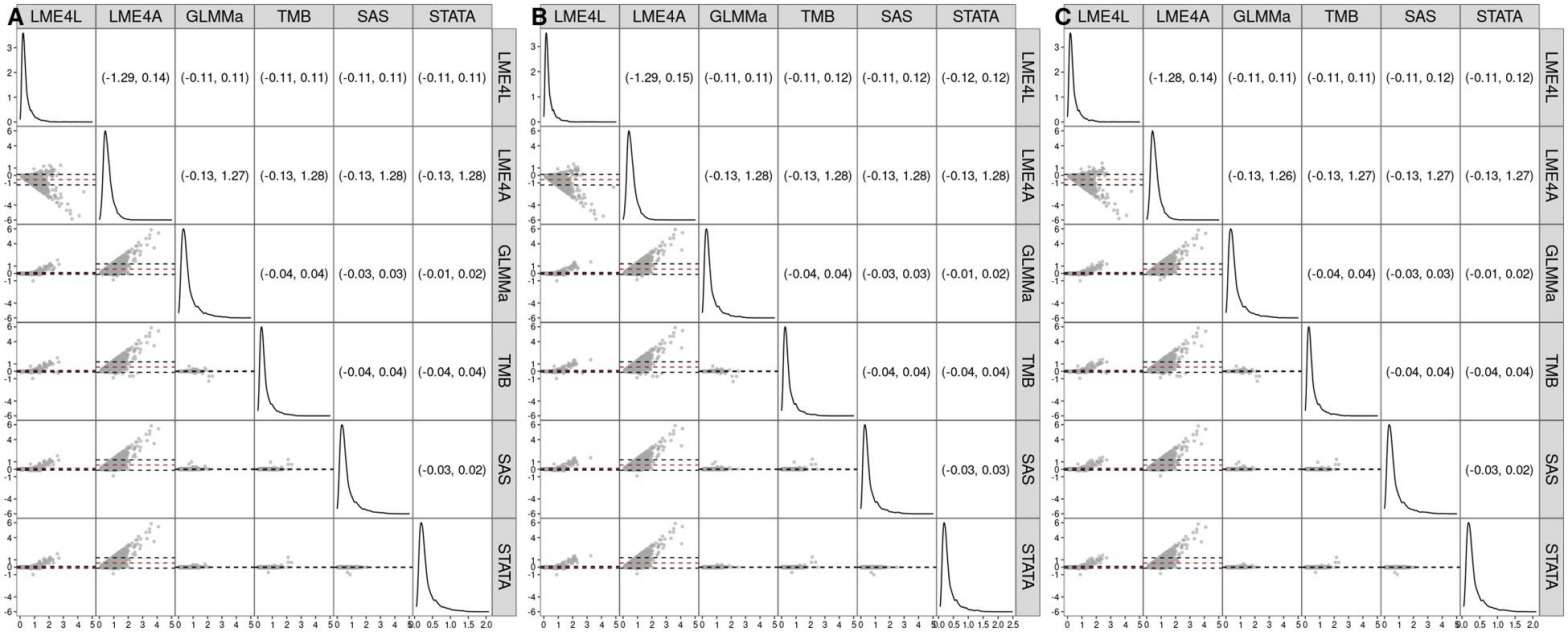
Estimated standard errors of the estimates of the mean-structure coefficients of the two-level model (19) for the different software implementations and the 9112 genes, for which the two-level model was successfully fitted for all the implementations. The densities of the estimated standard errors are shown along the diagonal. The lower triangle contains Bland–Altman plots of the estimates for pairs of different software implementations, with the red dashed line indicating the mean difference and the black dashed lines marking the 95% limits of agreement. The upper triangle presents the numerical values of the limits. Panel A: $\beta_{1}$; panel B: $\beta_{2}$; panel C: $\beta_{3}$. LME4L—**lme4** with the Laplace approximation; LME4A—**lme4** with the AGHQ approximation; GLMMa—**GLMMadaptive**; TMB—**glmmTMB**; SAS—**PROC NLMIXED**; STATA—**menbreg**.

Figure 3 presents the Bland–Altman plots and the 95% limits of agreement for the estimated standard errors of estimates of coefficients $\beta_{1}$ (Panel A), $\beta_{2}$ (Panel B), and $\beta_{3}$ (Panel C) for the different software implementations and the 9,112 genes, for which the two-level model (19) was successfully fitted for all the implementations. The mean difference of the standard errors is approximately 0 and there are no evident trends in plots for GLMMa, TMB, SAS, and STATA. The width of the 95% limits of agreement varies between 0.03 and 0.08, indicating a good agreement between the estimated standard errors (see also Table S5, available as supplementary data at *Bioinformatics Advances* online). As compared to those implementations, LME4L shows a trend toward larger estimates of larger standard errors, while the estimated standard errors for LME4A exhibit the most remarkable differences. In particular, Table S6, available as supplementary data at *Bioinformatics Advances* online, indicates that LME4A provides a larger estimate for over 98% of the 9112 genes, while for LME4L the percentage is considerably smaller (approximately 6%– 31%).

**Figure 4.**
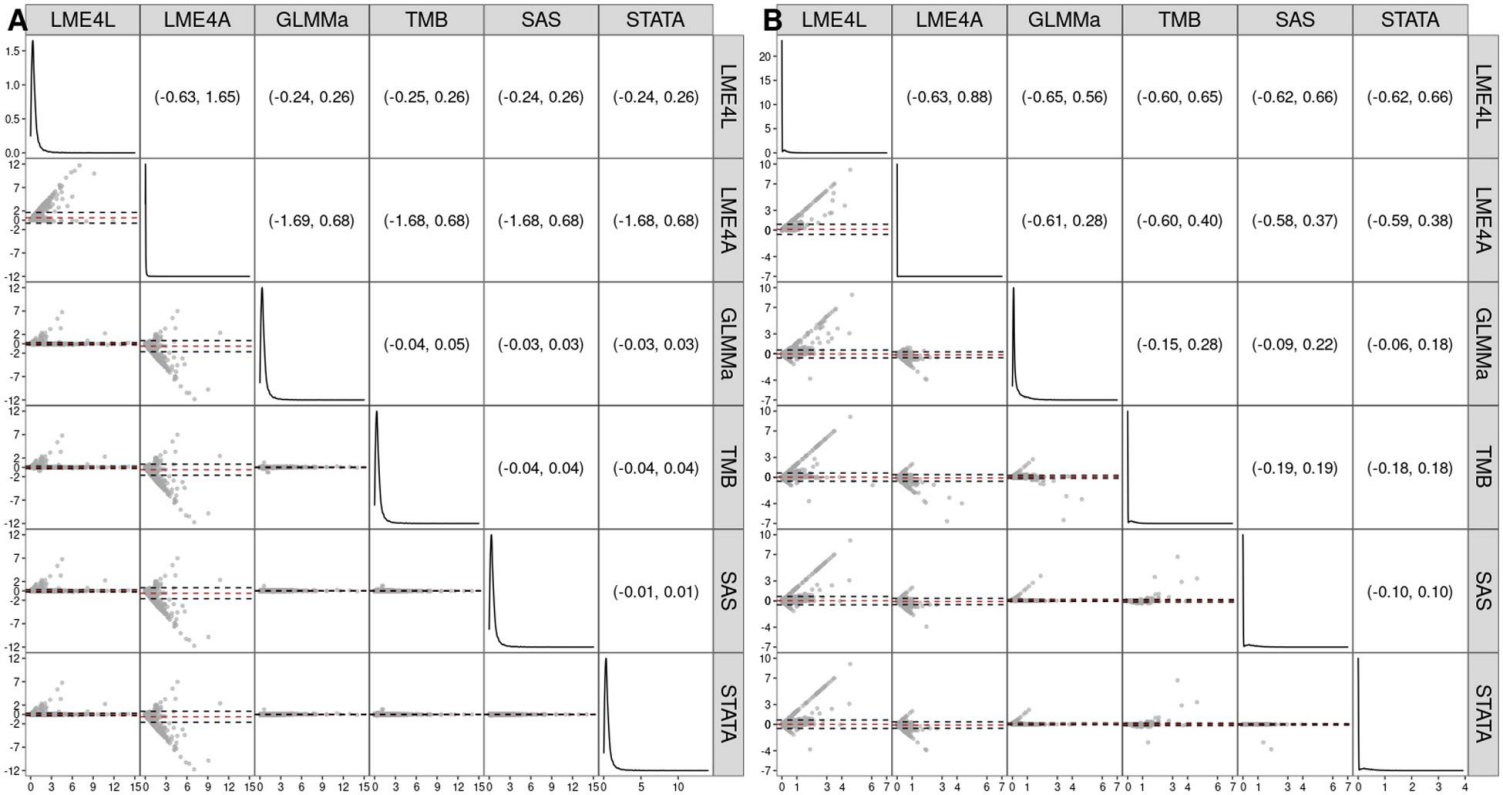
Estimates of ϕ and σ for the two-level model (19) for the different software implementations and the 9112 genes, for which the model was successfully fitted for all the implementations. The densities of the estimates are shown along the diagonal. The lower triangle contains Bland–Altman plots of the estimates for pairs of different software implementations, with the red dashed line indicating the mean difference and the black dashed lines marking the 95% limits of agreement. The upper triangle presents the numerical values of the 95% limits of agreement. Panel A: ϕ; panel B: σ. LME4L—**lme4** with the Laplace approximation; LME4A—**lme4** with the AGHQ approximation; GLMMa—**GLMMadaptive**; TMB—**glmmTMB**; SAS—**PROC NLMIXED**; STATA—**menbreg**.

Figure 4 presents the Bland-Altman plots and the 95% limits of agreement for the estimates of ϕ (Panel A) and σ (Panel B). For ϕ (Panel A), the estimated values for SAS, STATA, and GLMMa closely correspond to each other, as indicated by the location of the points along the horizontal red dashed line corresponding to the mean of 0 and the narrow limits of agreement (with the width no larger than 0.08). As compared to those implementations, LME4L and, especially, LME4A show marked deviations. For σ (Panel B), the estimates obtained by STATA, SAS, and TMB correspond, in general, closely to each other. As compared to those implementations, GLMMa sometimes produces larger estimates for small values of σ. The estimates provided by LME4A and LME4L markedly differ from the other four implementations.

Table 6 shows the results of testing the three null hypotheses of interest, $H_{0}^{123}$, $H_{0}^{1}$, and $H_{0}^{3}$ (see Section 2.1) for the 9112 genes for which the two-level model (19) was successfully fitted for all software implementations. In particular, the table presents, for each implementation, the number of genes for which a particular null hypothesis was rejected. The table contains the results obtained by using the uncorrected (“raw”) *P*-values for the chi-squared Wald-test statistics at the 5% significance level, as well as the *P*-values corrected for multiple testing by using the Benjamini-Hochberg procedure that controls the false discovery rate (FDR) at 5%. The results are quite consistent across the different software implementations except of LME4A. In particular, $H_{0}^{123}$ was rejected for approximately 40% of tested genes were rejected, suggesting that their expression for patients, as compared to healthy controls, was different at some of the three time points. For all implementations except LME4A, this conclusion was consistently reached by using the multiplicity-adjusted *P*-values for a set of 2853 genes. A similar pattern can be observed for the other two hypotheses, though with fewer rejections, i.e., approximately 18% and 21% for $H_{0}^{1}$ and $H_{0}^{3}$, respectively.

**Table 6.** The number of genes for which the particular null hypothesis was rejected (see Section 2.4) based on the two-level model (19).^a^

Hypothesis	Method	# genes (raw)	# genes (FDR)
$H_{0}^{123}$	LME4L	4023	2961
	LME4A	181	22
	GLMMa	3962	2883
	TMB	4015	2936
	SAS	4007	2927
	STATA	4007	2927
$H_{0}^{1}$	LME4L	1721	566
	LME4A	178	10
	GLMMa	1692	524
	TMB	1722	551
	SAS	1719	550
	STATA	1719	550
$H_{0}^{3}$	LME4L	2027	591
	LME4A	76	0
	GLMMa	1985	559
	TMB	2023	583
	SAS	2016	583
	STATA	2016	584

a The column marked “raw” contains the number of genes for which the null hypothesis was rejected at the 5% significance level without adjusting for multiple-testing. The column marked “FDR” contains the number of genes for which the null hypothesis was rejected after adjusting the *P*-values by using the Benjamini-Hochberg procedure. LME4L—**lme4** with the Laplace approximation; LME4A—**lme4** with the AGHQ approximation; GLMMa—**GLMMadaptive**; TMB—**glmmTMB**; SAS—**PROC NLMIXED**; STATA—**menbreg**.

As it is clear from Table 6, LME4A led to the smallest number of genes for which the null hypotheses could be rejected. The reason is the consistent overestimation of the standard errors, as compared to the other implementations, as noted in Fig. 3 and Table S6, available as supplementary data at *Bioinformatics Advances* online.

## 4 Conclusion

The hierarchical NB model allows analysis of over dispersed correlated count data resulting from experiments applying next-generation sequencing technologies, such as RNA-seq (Kazakiewicz *et al.* 2019, Tsonaka and Spitali 2021). Fitting the model requires the use of numerical integration and can be computationally intensive.

In this paper, we have evaluated several software implementations that can be used to apply the model. Table S1, available as supplementary data at *Bioinformatics Advances* online, provides a summary of packages and functions available in the open-source platform **R**, as well as in commercial software **SAS** and **STATA**. Toward this aim, we have used a real-life dataset obtained from an RNA-seq experiment. The estimates of the model parameters were quite consistent across different implementations.

Differences in estimates, particularly for σ and ϕ, as well as in computation times, which we observed in our paper, arise from several factors related to how different software implementations handle model fitting, parameterization, optimization, and numerical precision. To start with, the implementations use different approximations, such as the Laplace one or the AGHQ, to compute the marginal likelihood. Also, they use different optimization routines that can converge to different solutions. For instance, the Newton-Raphson method that was used in SAS is robust and accurate for complex likelihood functions, but tends to converge slower than other optimizers. LME4L and LME4A use, by default, derivative-free methods (such as Nelder-Mead or Bound Optimization by Quadratic Approximation), which can be less efficient in terms of speed and precision compared to gradient-based methods, especially in models with multiple random effects (Nocedal and Wright 2006).

In our evaluation, we often noted discrepancies between the results obtained by using the **LME4** package and the results for the other implementations. In the reference manual (Bates *et al.* 2015), the authors of the function glmer.nb, available in **LME4** for fitting models based on the NB distribution, indicates that certain components of the function are still experimental, with some methods still being either incomplete or suboptimal. This may explain the observed discrepancies. It is also worth noting that, unlike the other implementations, glmer.nb does not offer statistics that would allow inference about the dispersion parameter ϕ.

In general, the use of the AGHQ for the estimation of mixed-effects models is recommended, as compared to the use of the GHQ or the Laplace approximation (Bolker *et al.* 2009, Tsonaka and Spitali 2021). However, as compared to the Laplace approximation, the use of the AGHQ requires, in general, a longer computation time. In our case study, the estimates obtained by using the Laplace approximation were similar to the estimates obtained by using the AGHQ. Also, while it has been reported (Pinheiro and Bates 1995, Lesaffre and Spiessens 2001) that the number of quadrature points in the AGHQ approximation can substantially impact the estimation of a mixed-effects model, we have not observed any substantial differences in our results when increasing the number of the points beyond 10. There is no definitive guideline for the optimal number of quadrature points to use in practice, but using between 5 and 10 has been shown as a good balance between precision and computational efficiency (Pinheiro and Bates 1995, Lesaffre and Spiessens 2001, Pinheiro and Chao 2006). Software implementations also vary in their defaults. For example, **GLMMadaptive** typically uses 11 quadrature points when there are one or two random effects, and 7 points otherwise. In contrast, **LME4** recommends that models with a single random effect can reasonably use up to 25 quadrature points. Nevertheless, we recommend that the decision regarding the number of the points to be used should be made on a case-by-case basis.

The data used for illustration in this paper were obtained from a longitudinal study that included 42 subjects (Bouquet *et al.* 2016). In practice, smaller sample sizes can be encountered. In that case, it is recommended that statistical hypothesis testing is conducted by using bootstrap (Tsonaka and Spitali 2021). A drawback of the use of bootstrap is an increase in the computation time necessary to conduct an analysis.

The various software implementations, evaluated in our paper, allow for a routine use of the hierarchical NB model in analysis of genomic experiments. We believe that the presented results may help the researchers to choose an appropriate implementation. To further assist in that matter, we include the code, used to fit models (18) and (19) to the data for the ENSG00000144802 gene by applying the different implementations, on Zenodo (https://doi.org/10.5281/zenodo.14908112).

## Supplementary Material

vbaf126_Supplementary_Data

## Data Availability

The data underlying this article are available on Zenodo (https://doi.org/10.5281/zenodo.14908112).

## Author Biographies

**Katarzyna Górczak** is a PhD student in Statistics at the Data Science Institute, Hasselt University. Her research focuses on statistical methods for “omics” data.

**Tomasz Burzykowski** is Professor of Biostatistics and Bioinformatics at the Data Science Institute, Hasselt University. His research focuses on statistical methods in clinical research and “omics” experiments.

**Jürgen Claesen** is Assistant Professor at the Department of Epidemiology and Data Science at the Amsterdam UMC, and guest lecturer at the Data Science Institute, Hasselt University. His research focuses on statistical methods for mass spectrometry, sequencing, and structural biology.
