# Supplementary material for: A hierarchical negative-binomial model for analysis of correlated sequencing data: practical implementations
Source: Bioinform Adv. 2025 Jun 10;5(1):vbaf126. doi: 10.1093/bioadv/vbaf126 (PMC12256765; doi:10.1093/bioadv/vbaf126)
Supplement: vbaf126_Supplementary_Data [file vbaf126_Supplementary_Data.zip › Supplementary Material.pdf]

Supplementary material

Additional tables

Software	Package	Function /command	Integral approximation	Random-effects structure
	`LME4`	`glmer.nb`	Laplace	multiple-level (nested or crossed)
		`glmer.nb`	AGHQ	two-level
**R**	`TMB`	`glmmTMB`	Laplace	multiple-level (nested or crossed)
	`ADMB`	`glmmadmb`	Laplace	multiple-level (nested or crossed)
	`GLMMadaptive`	`mixed_model`	AGHQ	two-level
**STATA**	-	`menbreg`	AGHQ	multiple-level (nested or crossed)
**SAS**	-	`NLMIXED`	AGHQ	multiple-level (nested)

Table S1: Summary of various software implementations. AGHQ - adaptive Gauss-Hermite quadrature.

		Method				
Param	Coef	`LME4L`	`TMB`	`ADMB`	`STATA`	`SAS`
β_0	est	-15.1390	-15.4359	-15.4359	-15.4384	-15.4360
	se	0.1554	0.1507	0.1507	0.1479	0.1508
β_1	est	0.7230	0.7495	0.7495	0.7520	0.7496
	se	0.1884	0.1816	0.1816	0.1787	0.1816
β_2	est	0.5330	0.5151	0.5151	0.5178	0.5154
	se	0.1888	0.1832	0.1832	0.1784	0.1832
β_3	est	0.1478	0.3254	0.3254	0.3281	0.3257
	se	0.1875	0.1822	0.1822	0.1813	0.1822
σ_S	est	0.5062	0.2297	0.2297	0.2328	0.2321
	se	0.0472	0.0891	0.0891	0.0890	0.0886
σ_I	est	0.2537	0.2262	0.2262	0.2234	0.2236
	se	0.0859	0.1063	0.1063	0.1075	0.1076
ϕ	est	26429.5492	0.5112	0.5112	0.5112	0.5112
	se	-	0.0199	0.0199	0.0199	0.0199
Time (s)		7.78	1.97	71.42	308.46	378.90

Table S2: Estimates ('est') of the coefficients, standard errors ('se') and computational time for the three-level model (18), see Section 2.1. Gene ENSG00000144802. `LME4L` - **lme4** with Laplace approximation; `TMB` - **glmmTMB**; `ADMB` - **glmmadmb**; `SAS` - **PROC NLMIXED**; `STATA` - **menbreg**. For the AGHQ-based methods, 75 quadrature points were used.

method	# fit succeeded	# fit failed	# fit failed (A)	# fit failed (B)	# fit failed (C)
LME4L	10832	7933	2002	5924	7
LME4A	11754	7011	1844	5162	5
GLMMa	15997	2768	2731	0	37
TMB	16449	2316	0	1863	453
SAS	15820	2945	222	0	2723
STATA	14732	4033	2936	1097	0

Table S3: The number of genes (out of 18,765) for which the two-level model (1)–(3) was successfully fitted. Columns: A - cases for which no results were obtained; B - cases for which results were obtained with warnings related to convergence, large or not uniquely determined parameters, etc.; C - cases for which variance-covariance matrix was not positive-definite or contained missing values. `LME4L` - **lme4** with the Laplace approximation; `LME4A` - **lme4** with the AGHQ approximation; `GLMMa` - **GLMMadaptive**; `TMB` - **glmmTMB**; `SAS` - **PROC NLMIXED**; `STATA` - **menbreg**.

	β_1		β_2		β_3	
methods	mean	95% loa	mean	95% loa	mean	95% loa
LME4A - GLMMa	-0.0024	(-0.3480, 0.3431)	-0.0056	(-0.3526, 0.3414)	-0.0039	(-0.3384, 0.3306)
LME4A - TMB	-0.0019	(-0.3461, 0.3423)	-0.0044	(-0.3508, 0.3420)	-0.0023	(-0.3354, 0.3308)
LME4A - SAS	-0.0020	(-0.3473, 0.3434)	-0.0047	(-0.3514, 0.3420)	-0.0026	(-0.3361, 0.3308)
LME4A - STATA	-0.0021	(-0.3485, 0.3443)	-0.0049	(-0.3531, 0.3432)	-0.0029	(-0.3386, 0.3327)
LME4L - LME4A	0.0013	(-0.3603, 0.3629)	0.0041	(-0.3542, 0.3623)	0.0034	(-0.3420, 0.3488)
LME4L - GLMMa	-0.0011	(-0.1053, 0.1031)	-0.0015	(-0.0890, 0.0859)	-0.0005	(-0.1046, 0.1036)
LME4L - TMB	-0.0006	(-0.1219, 0.1208)	-0.0003	(-0.1076, 0.1070)	0.0011	(-0.1221, 0.1243)
LME4L - SAS	-0.0006	(-0.1218, 0.1205)	-0.0006	(-0.1071, 0.1059)	0.0008	(-0.1208, 0.1223)
LME4L - STATA	-0.0008	(-0.1202, 0.1186)	-0.0008	(-0.1061, 0.1044)	0.0005	(-0.1210, 0.1220)
GLMMa - TMB	0.0006	(-0.0541, 0.0552)	0.0012	(-0.0573, 0.0598)	0.0016	(-0.0566, 0.0599)
GLMMa - SAS	0.0005	(-0.0505, 0.0515)	0.0009	(-0.0545, 0.0563)	0.0013	(-0.0589, 0.0614)
GLMMa - STATA	0.0003	(-0.0446, 0.0452)	0.0007	(-0.0461, 0.0475)	0.0010	(-0.0472, 0.0491)
TMB - SAS	-0.0001	(-0.0241, 0.0239)	-0.0003	(-0.0321, 0.0315)	-0.0004	(-0.0335, 0.0328)
TMB - STATA	-0.0003	(-0.0306, 0.0300)	-0.0005	(-0.0351, 0.0341)	-0.0006	(-0.0328, 0.0315)
SAS - STATA	-0.0002	(-0.0245, 0.0242)	-0.0002	(-0.0299, 0.0295)	-0.0003	(-0.0364, 0.0359)

Table S4: The mean difference and the 95% limits of agreement (loa) of the estimates of the mean-structure coefficients for the two-level model (19). The mean and limits are calculated for each par of methods. `LME4L` - **lme4** with the Laplace approximation; `LME4A` - **lme4** with the AGHQ approximation; `GLMMa` - **GLMMadaptive**; `TMB` - **glmmTMB**; `SAS` - **PROC NLMIXED**; `STATA` - **menbreg**.

	β_1		β_2		β_3	
methods	mean	95% loa	mean	95% loa	mean	95% loa
LME4A - GLMMa	0.5707	(-0.1330, 1.2744)	0.5727	(-0.1334, 1.2788)	0.5660	(-0.1322, 1.2642)
LME4A - TMB	0.5732	(-0.1307, 1.2770)	0.5751	(-0.1313, 1.2815)	0.5684	(-0.1299, 1.2667)
LME4A - SAS	0.5730	(-0.1301, 1.2761)	0.5749	(-0.1307, 1.2805)	0.5682	(-0.1294, 1.2658)
LME4A - STATA	0.5728	(-0.1306, 1.2762)	0.5747	(-0.1313, 1.2807)	0.5680	(-0.1299, 1.2659)
LME4L - LME4A	-0.5709	(-1.2857, 0.1438)	-0.5727	(-1.2907, 0.1452)	-0.5661	(-1.2757, 0.1435)
LME4L - GLMMa	-0.0002	(-0.1074, 0.1069)	-0.0001	(-0.1126, 0.1125)	-0.0001	(-0.1091, 0.1089)
LME4L - TMB	0.0022	(-0.1072, 0.1116)	0.0024	(-0.1113, 0.1160)	0.0023	(-0.1085, 0.1131)
LME4L - SAS	0.0020	(-0.1097, 0.1138)	0.0022	(-0.1139, 0.1182)	0.0021	(-0.1110, 0.1152)
LME4L - STATA	0.0019	(-0.1109, 0.1146)	0.0020	(-0.1151, 0.1190)	0.0019	(-0.1121, 0.1159)
GLMMa - TMB	0.0025	(-0.0394, 0.0443)	0.0024	(-0.0394, 0.0442)	0.0024	(-0.0389, 0.0437)
GLMMa - SAS	0.0023	(-0.0280, 0.0325)	0.0022	(-0.0268, 0.0313)	0.0022	(-0.0273, 0.0317)
GLMMa - STATA	0.0021	(-0.0149, 0.0191)	0.0020	(-0.0125, 0.0166)	0.0020	(-0.0140, 0.0180)
TMB - SAS	-0.0002	(-0.0409, 0.0405)	-0.0002	(-0.0418, 0.0414)	-0.0002	(-0.0407, 0.0403)
TMB - STATA	-0.0004	(-0.0388, 0.0380)	-0.0004	(-0.0397, 0.0389)	-0.0004	(-0.0386, 0.0379)
SAS - STATA	-0.0002	(-0.0253, 0.0249)	-0.0002	(-0.0254, 0.0250)	-0.0002	(-0.0250, 0.0247)

Table S5: The mean difference and the 95% limits of agreement (loa) of the estimated standard errors of the mean-structure coefficients for the two-level model (19). The mean and limits are calculated for each par of methods. `LME4L` - **lme4** with the Laplace approximation; `LME4A` - **lme4** with the AGHQ approximation; `GLMMa` - **GLMMadaptive**; `TMB` - **glmmTMB**; `SAS` - **PROC NLMIXED**; `STATA` - **menbreg**.

	LME4A			LME4L		
	β_1	β_2	β_3	β_1	β_2	β_3
LME4L	98.41	98.35	98.36	-	-	-
LME4A	-	-	-	1.59	1.65	1.64
GLMMa	98.85	98.83	98.84	6.08	6.01	6.04
TMB	98.99	98.94	98.98	18.56	18.67	19.85
SAS	98.94	98.90	98.95	30.68	30.34	31.05
STATA	98.91	98.89	98.92	23.13	22.75	23.38

Table S6: The percentage of genes (out of 9,112) for which LME4A and LME4L yield a higher standard error for the three coefficients of interest (β_1, β_2 and β_3), as compared to the other implementations. LME4L - **lme4** with the Laplace approximation; LME4A - **lme4** with the AGHQ approximation; GLMMa - **GLMMadaptive**; TMB - **glmmTMB**; SAS - **PROC NLMIXED**; STATA - **menbreg**.
